# Author Correction: Perceptions of the appropriate response to norm violation in 57 societies

**DOI:** 10.1038/s41467-021-22955-x

**Published:** 2021-04-26

**Authors:** Kimmo Eriksson, Pontus Strimling, Michele Gelfand, Junhui Wu, Jered Abernathy, Charity S. Akotia, Alisher Aldashev, Per A. Andersson, Giulia Andrighetto, Adote Anum, Gizem Arikan, Zeynep Aycan, Fatemeh Bagherian, Davide Barrera, Dana Basnight-Brown, Birzhan Batkeyev, Anabel Belaus, Elizaveta Berezina, Marie Björnstjerna, Sheyla Blumen, Paweł Boski, Fouad Bou Zeineddine, Inna Bovina, Bui Thi Thu Huyen, Juan-Camilo Cardenas, Đorđe Čekrlija, Hoon-Seok Choi, Carlos C. Contreras-Ibáñez, Rui Costa-Lopes, Mícheál de Barra, Piyanjali de Zoysa, Angela Dorrough, Nikolay Dvoryanchikov, Anja Eller, Jan B. Engelmann, Hyun Euh, Xia Fang, Susann Fiedler, Olivia A. Foster-Gimbel, Márta Fülöp, Ragna B. Gardarsdottir, C. M. Hew D. Gill, Andreas Glöckner, Sylvie Graf, Ani Grigoryan, Vladimir Gritskov, Katarzyna Growiec, Peter Halama, Andree Hartanto, Tim Hopthrow, Martina Hřebíčková, Dzintra Iliško, Hirotaka Imada, Hansika Kapoor, Kerry Kawakami, Narine Khachatryan, Natalia Kharchenko, Ninetta Khoury, Toko Kiyonari, Michal Kohút, Lê Thuỳ Linh, Lisa M. Leslie, Yang Li, Norman P. Li, Zhuo Li, Kadi Liik, Angela T. Maitner, Bernardo Manhique, Harry Manley, Imed Medhioub, Sari Mentser, Linda Mohammed, Pegah Nejat, Orlando Nipassa, Ravit Nussinson, Nneoma G. Onyedire, Ike E. Onyishi, Seniha Özden, Penny Panagiotopoulou, Lorena R. Perez-Floriano, Minna S. Persson, Mpho Pheko, Anna-Maija Pirttilä-Backman, Marianna Pogosyan, Jana Raver, Cecilia Reyna, Ricardo Borges Rodrigues, Sara Romanò, Pedro P. Romero, Inari Sakki, Alvaro San Martin, Sara Sherbaji, Hiroshi Shimizu, Brent Simpson, Erna Szabo, Kosuke Takemura, Hassan Tieffi, Maria Luisa Mendes Teixeira, Napoj Thanomkul, Habib Tiliouine, Giovanni A. Travaglino, Yannis Tsirbas, Richard Wan, Sita Widodo, Rizqy Zein, Qing-peng Zhang, Lina Zirganou-Kazolea, Paul A. M. Van Lange

**Affiliations:** 1 Center for Cultural Evolution, Stockholm University, Stockholm, Sweden; 2 Mälardalen University, Västerås, Sweden; 3 Institute for Futures Studies, Box 591, Stockholm, Sweden; 4 Department of Psychology, University of Maryland, College Park, MD USA; 5 CAS Key Laboratory of Behavioral Sciences, Chinese Academy of Sciences, Chaoyang District, Beijing, China; 6 Department of Sociology, University of South Carolina, Columbia, SC USA; 7 Department of Psychology, University of Ghana, P.O. Box LG 84 Legon, Accra, Ghana; 8 New School of Economics, Satbayev University, Almaty, Kazakhstan; 9 Department of Behavioral Sciences and Learning, Linköping University, Linköping, Sweden; 10 Institute of Cognitive Sciences and Technologies, National Research Council of Italy, Rome, Italy; 11 Department of Political Science, Trinity College Dublin, Dublin 2, Ireland; 12 Koç University, Rumelifeneri, Sarıyer Rumelifeneri Yolu, Istanbul, Turkey; 13 Department of Psychology and Education, Shahid Beheshti University, Tehran, Iran; 14 University of Turin and Collegio Carlo Alberto, Turin, Italy; 15 United States International University – Africa, Box 14634 00800, Nairobi, Kenya; 16 International School of Economics, Kazakh-British Technical University, Almaty, Kazakhstan; 17 Instituto de Investigaciones Psicológicas (IIPsi), Consejo Nacional de Investigaciones Científicas y Técnicas (CONICET), CABA, República Argentina; 18 Universidad Nacional de Córdoba (UNC). Facultad de Psicología (UNC), Ciudad Universitaria, Bv. de la Reforma esquina, Enfermera Gordillo s/n, Córdoba, Argentina; 19 Sunway University, No. 5, Jalan Universiti, Bandar Sunway, Selangor, Darul Ehsan Malaysia; 20 Departamento de Psicología, Pontificia Universidad Católica del Perú, San Miguel, Lima Peru; 21 SWPS University of Social Sciences and Humanities, Warsaw, Chodakowska Poland; 22 Department of Psychology, University of Innsbruck, Innsbruck, Austria; 23 Moscow State University of Psychology and Education, Moscow, Russia; 24 Hanoi National University of Education, Cau Giay District, Hanoi Vietnam; 25 Universidad de los Andes, Colombia, Bogota, Colombia; 26 Faculty of philosophy, University of Banja Luka, Banja Luka, Bosnia and Herzegovina; 27 Department of Psychology, Sungkyunkwan University, Seoul, Republic of Korea; 28 Departamento de Sociología, Universidad Autónoma Metropolitana - Unidad Iztapalapa, Ciudad de México, Mexico; 29 Instituto de Ciências Sociais, Universidade de Lisboa, Lisboa, Portugal; 30 Center for Culture and Evolution, Brunel University London, Uxbridge, UK; 31 Faculty of Medicine, University of Colombo, Colombo 8, Sri Lanka; 32 Department of Psychology, University of Cologne, Cologne, Germany; 33 Facultad de Psicología, Universidad Nacional Autónoma de México. Av. Universidad 3004, Ciudad Universitaria, Ciudad de México, Mexico; 34 Center for Research in Experimental Economics and Political Decision Making (CREED), Amsterdam School of Economics, University of Amsterdam, P.O. Box 15867, Amsterdam, NJ The Netherlands; 35 Department of Psychology, University of Minnesota, Minneapolis, MN 55455 USA; 36 Department of Psychology, York University, Toronto, ON Canada; 37 Max Planck Institute for Research on Collective Goods, Bonn, Germany; 38 New York University, Stern School of Business, New York, NY 10012 USA; 39 Institute for Cognitive Neuroscience and Psychology, Research Centre of Natural Sciences, Budapest, Hungary; 40 Eötvös Loránd University, Faculty of Psychology and Education, Budapest, Hungary; 41 Department of Psychology, University of Iceland, Reykjavík, Iceland; 42 Institute of Psychology, Czech Academy of Sciences, Brno, Czech Republic; 43 Department of Personality Psychology, Yerevan State University, Yerevan, Armenia; 44 Saint Petersburg State University, St Petersburg, Russia; 45 Center for Social and Psychological Sciences, Slovak Academy of Sciences, Bratislava, Slovakia; 46 School of Social Sciences, Singapore Management University, Singapore, Singapore; 47 School of Psychology, University of Kent, Canterbury, UK; 48 Daugavpils University, Daugvapils, Latvia; 49 Department of Psychology, Mumbai, Maharashtra India; 50 Kyiv International Institute of Sociology, Kyiv, Ukraine; 51 Future Minds Gifted Centre, Lima, Peru; 52 Aoyama Gakuin University, Sagamihara-city, Kanagawa Japan; 53 Faculty of Philosophy and Arts, University of Trnava, Trnava, Slovakia; 54 National Economics University, Hai Ba Trung, Dong Tam District, Hanoi, Vietnam; 55 Nagoya University, Furo-cho, Chikusa-ku, Nagoya, Aichi Japan; 56 Melbourne School of Psychological Science, University of Melbourne, Parkville, VIC Australia; 57 Department of Psychology, University of Western Ontario, London, ON Canada; 58 School of Natural Sciences and Health, Tallinn University, Tallinn, Estonia; 59 Department of International Studies, American University of Sharjah, PO Box 26666, Sharjah, United Arab Emirates; 60 Eduardo Mondlane University, Faculty of Arts and Social Sciences, Department of Sociology, Maputo, Mozambique; 61 Faculty of Psychology, Chulalongkorn University, Bangkok, Thailand; 62 Department of Finance and Investment, Al Imam Mohammad Ibn Saud Islamic University (IMSIU), P.O. Box 5701, Riyadh, Saudi Arabia; 63 The Open University of Israel, Raanana, Israel; 64 Institute of Criminology and Public Safety, Valsayn Campus, Graver Road, Valsayn, University of Trinidad and Tobago, Arima, Trinidad and Tobago; 65 University of Haifa, Haifa, Israel; 66 Department of Psychology, University of Nigeria Nsukka, Nsukka, Nigeria; 67 Department of Education and Social Work, University of Patras, Rion, Patras Greece; 68 Universidad Diego Portales, Santiago, Chile; 69 Department of Psychology, University of Botswana, Private Bag UB 00705, Gaborone, Botswana; 70 University of Helsinki, Faculty of Social Sciences, Social Psychology, PO Box 54 (Unioninkatu 37), Helsinki, Finland; 71 Politics, Psychology, Law and Economics (PPLE), University of Amsterdam, PO Box 15575, Amsterdam, The Netherlands; 72 Queen’s University, Goodes Hall, Queen’s University, Kingston, ON Canada; 73 Instituto Universitário de Lisboa ISCTE-IUL, CIS, Lisbon, Portugal; 74 Department of Culture, Politics and Society, University of Turin, Turin, Italy; 75 Experimental and Computational Economics Lab (ECEL), School of Economics, Universidad San Francisco de Quito, Diego de Robles y Pampite, Quito, Ecuador; 76 University of Eastern Finland, Department of Social Sciences, P.O. Box 162770211, Kuopio, Finland; 77 IESE Business School, Madrid, Spain; 78 Kwansei Gakuin University, Nishinomiya, Hyogo Japan; 79 Department of International Management, Johannes Kepler University, Linz, Austria; 80 Faculty of Economics, Shiga University, Hikone, Shiga Japan; 81 Université Félix Houphouët-Boigny Cocody-Abidjan, Centre Ivoirien d’Etude et de Recherche en Psychologie Appliquée (CIERPA), Abidjan, Côte d’Ivoire; 82 Mackenzie Presbyterian University, Business Administration Postgraduate Program, São Paulo, Brazil; 83 Labo-PECS, Faculty of Social Sciences, Université d’Oran 2, Oran, Algeria; 84 School of Humanities and Social Science, The Chinese University of Hong Kong, Shenzhen, Longgang District, Shenzhen P. R. China; 85 University of Athens, Department of Political Science and Public Administration, Athens, Greece; 86 Department of Personality and Social Psychology, Universitas Airlangga, Surabaya, Indonesia; 87 Guangzhou University, Guangzhou Higher Education Mega Center, Guangzhou, P.R. China; 88 VU Amsterdam, Department of Experimental and Applied Psychology, Institute for Brain and Behavior Amsterdam (IBBA), Amsterdam, Amsterdam, The Netherlands

**Keywords:** Human behaviour, Psychology and behaviour

Correction to: *Nature Communications* 10.1038/s41467-021-21602-9, published online 5 March 2021.

The original version of this Article contained an error in the author affiliations.

Cecilia Reyna was incorrectly associated with ‘Universidad Nacional de Córdoba (UNC). Facultad de Psicología (UNC), Ciudad Universitaria, Bv. de la Reforma esquina, Enfermera Gordillo s/n, Córdoba, Argentina.’ instead of the correct ‘Instituto de Investigaciones Psicológicas (IIPsi), Consejo Nacional de Investigaciones Científicas y Técnicas (CONICET), CABA, República Argentina.’

This has now been corrected in both the PDF and HTML versions of the Article.

