# Supplementary material for: Perceptions of the appropriate response to norm violation in 57 societies
Source: Nat Commun. 2021 Mar 5;12:1481. doi: 10.1038/s41467-021-21602-9 (PMC7935962; doi:10.1038/s41467-021-21602-9)
Supplement: Supplementary file 1 — Supplementary Information [file 41467_2021_21602_MOESM1_ESM.pdf]

Supplementary Information

for Perceptions of the appropriate response to norm violation in 57 Societies

Kimmo Eriksson*, Pontus Strimling, Michele Gelfand, Junhui Wu, Jered Abernathy, Charity S. Akotia, Alisher Aldashev, Per A. Andersson, Giulia Andrighetto, Adote Anum, Gizem Arikan, Zeynep Aycan, Fatemeh Bagherian, Davide Barrera, Dana Basnight-Brown, Birzhan Batkeyev, Anabel Belaus, Elizaveta Berezina, Marie Björnstjerna, Sheyla Blumen, Paweł Boski, Fouad Bou Zeineddine, Inna Bovina, Bui Thi Thu Huyen, Juan-Camilo Cardenas, Đorđe Čekrlija, Hoon-Seok Choi, Carlos C. Contreras-Ibáñez, Rui Costa-Lopes, Mícheál de Barra, Piyanjali de Zoysa, Angela Dorrough, Nikolay Dvoryanchikov, Anja Eller, Jan B. Engelmann, Hyun Euh, Xia Fang, Susann Fiedler, Olivia A. Foster-Gimbel, Márta Fülöp, Ragna B. Gardarsdottir, C. M. Hew D. Gill, Andreas Glöckner, Sylvie Graf, Ani Grigoryan, Vladimir Gritskov, Katarzyna Growiec, Peter Halama, Andree Hartanto, Tim Hopthrow, Martina Hřebíčková, Dzintra Iliško, Hirotaka Imada, Hansika Kapoor, Kerry Kawakami, Narine Khachatryan, Natalia Kharchenko, Ninetta Khoury, Toko Kiyonari, Michal Kohút, Linh Thuy Le, Lisa M. Leslie, Yang Li, Norman P. Li, Zhuo Li, Kadi Liik, Angela T. Maitner, Bernardo Manhique, Harry Manley, Imed Medhioub, Sari Mentser, Linda Mohammed, Pegah Nejat, Orlando Nipassa, Ravit Nussinson, Nneoma G. Onyedire, Ike E. Onyishi, Seniha Özden, Penny Panagiotopoulou, Lorena R. Perez-Floriano, Minna Persson, Mpho Pheko, Anna-Maija Pirttilä-Backman, Marianna Pogosyan, Jana Raver, Cecilia Reyna, Ricardo Borges Rodrigues, Sara Romanò, Pedro P. Romero, Inari Sakki, Alvaro San Martin, Sara Sherbaji, Hiroshi Shimizu, Brent Simpson, Erna Szabo, Kosuke Takemura, Hassan Tieffi, Maria Luisa Mendes Teixeira, Napoj Thanomkul, Habib Tiliouine, Giovanni A. Travaglino, Yannis Tsirbas, Richard Wan, Sita Widodo, Rizqy Zein, Qing-peng Zhang, Lina Zirganou-Kazolea, Paul A.M. Van Lange

Correspondence to: kimmoe@gmail.com

This PDF file includes Supplementary Tables 1-9 and Supplementary Figure 1

Supplementary Table 1. Description of sample characteristics of country, data collection site(s), language of survey, student sample (size, age, gender), and non-student sample (size, age, gender).

			Student sample			Non-student sample		
Country	Survey language	City	Size	Mean age	% Female	Size	Mean age	% Female
Algeria	Arabic	Oran	82	23.2	78.6			
Argentina	Spanish, South American*	Córdoba	214	21.8	69.6	478	29.5	66.0
Armenia	Armenian	Yerevan	406	21.8	67.4	157	29.1	62.5
Australia	English	Melbourne	255	19.6	75.5			
Austria	German	Linz	360	22.0	66.2			
Bosnia and Herzegovina	Bosnian-Croatian-Serbian	Banja Luka	289	21.2	49.8			
Botswana	English	Gaborone	102	20.9	82.2			
Brazil	Brazilian Portuguese	Saõ Paolo	262	27.8	51.0	124	39.9	61.1
Canada	English	Kingston	210	19.8	55.6			
		Toronto	231	20.8	77.5			
Chile	Spanish, South American*	Santiago	159	20.9	42.3			
China	Chinese simplified	Beijing	289	19.9	81.1	138	30.2	42.4
		Guangzhou	233	18.9	71.0	121	37.2	57.8
		Shenzhen	622	20.2	67.2			
Colombia	Spanish, South American*	Bogotá	231	20.3	53.9	119	36.5	73.5
Czech Republic	Czech	Brno	360	25.6	75.6	188	34.3	77.5
Ecuador	Spanish, South American*	Quito	328	21.5	62.2	53	28.8	59.1
Estonia	Estonian	Tallinn	287	28.1	75.7	120	32.3	85.0
Finland	Finnish	Helsinki	356	28.7	76.0	38	49.8	72.0
Germany	German	Cologne	204	23.0	60.1	98	31.6	47.9
		Hagen				413**	31.5	77,5
Ghana	English	Accra	220	20.8	62.1	60	31.3	40.0
Greece	Greece	Athens	160	26.1	55.3	28	37.1	52.9
		Patras	231	21.6	78.7	151	38.2	66.9

Hungary	Hungarian	Budapest	356	21.6	76.4	96	34.6	89.6
Iceland	Icelandic	Reykjavik	834	27.0	74.3	190	44.2	76.9
India	English	Mumbai	283	18.9	87.8	31	37.0	38.5
Indonesia	Bahasa Indonesia	Surabaya	212	19.7	86.6	120	27.6	71.1
Iran	Farsi	Teheran	230	21.5	71.6			
Ireland	English	Dublin	247	20.9	57.1	23	43.6	69.2
Israel	Hebrew	Tel Aviv	265	26.6	68.7	127	30.3	41.3
Italy	Italian	Rome	189	22.3	43.1			
		Turin	212	23.4	70.0			
Ivory Coast	French	Abidjan	270	26.2	42.4			
Japan	Japanese	Kanagawa	265	20.1	42.9			
		Osaka-Kobe	309	19.7	60.7			
Kazakhstan	Russian	Almaty	201	19.7	58.2			
Kenya	English	Nairobi	196	22.0	48.4			
Latvia	Latvian	Daugavpils	436	28.7	71.8	66	38.4	58.3
Malaysia	English	Kuala Lumpur	290	21.5	48.8	122	33.4	58.1
Mexico	Spanish. South American*	Mexico City	222	25.2	68.1			
Mozambique	Portuguese	Maputo	250	23.1	34.3			
Netherlands	English	Amsterdam	379	21.7	54.8			
Nigeria	English	Nsukka	240	22.7	58.2	217	36.1	54.2
Peru	Spanish. South American*	Lima	126	29.3	68.6	240	36.8	66.9
Poland	Polish	Warsaw	283	27.0	83.2	359	41.8	60.4
Portugal	Portuguese	Lisbon	227	24.3	88.7			
Qatar	Arabic	Doha	62	28.4	85.2	59	29.1	89.2
Russia	Russian	Moscow	382	23.0	76.5			
		St Petersburg	216	21.9	73.6			
Saudi Arabia	Arabic	Riyadh	261	21.5	11.4	95	32.8	58.4
Singapore	English	Singapore	205	22.0	68.3			
Slovakia	Slovak	Bratislava	363	25.6	61.9	124	39.0	43.8
South Korea	Korean	Seoul	252	20.8	52.7	135	39.5	55.2
Spain	Spanish	Madrid	134	19.5	50.4	164	42.3	35.0
Sri Lanka	English	Colombo	377	23.2	60.6			
Sweden	Swedish	Linköping	165	24.7	53.9			
		Stockholm	40	37.4	50.0			

Thailand	Thai	Bangkok	228	19.8	67.9			
Trinidad and Tobago	English	Port of Spain	173	24.7	74.4	73**	29.0	85.7
Turkey	Turkish	Istanbul	269	21.6	83.4			
United Arab Emirates	English	Sharjah	307	20.0	63.8			
Ukraine	Ukrainian, Russian	Kiev	447	22.9	70.7	118	38.5	55.4
United Kingdom	English	Canterbury	193	19.7	80.2			
		London	122	19.1	89.1	127	36.4	85.4
United States	English	Columbia. SC	497	19.4	76.8			
		New York	217	21.9	74.3			
Vietnam	Vietnamese	Hanoi	568	18.7	75.8			
Total: 57	30	68	18091			4772		

* With a few local lexical changes.
** Part-time students.

Supplementary Table 2. Metanorm measures.

	Metanorms for				
Country	physical confrontation	verbal confrontation	social ostracism	gossip	non-action
Algeria	2.57	2.69	2.27	2.02	2.63
Argentina	1.55	2.80	1.89	2.37	2.63
Armenia	2.59	2.80	2.03	2.07	2.76
Australia	1.51	2.37	1.95	2.68	2.76
Austria	1.38	2.61	1.97	2.48	2.70
Bosnia&Herzegovina	2.36	2.85	2.09	1.87	2.84
Botswana	1.85	2.42	2.08	2.45	2.64
Brazil	1.68	2.76	1.98	2.26	2.74
Canada	1.51	2.25	2.07	2.65	2.74
Chile	1.82	2.90	1.98	2.33	2.52
China	2.19	2.51	2.20	2.42	2.66
Colombia	1.54	2.84	1.86	2.43	2.61
Czech Republic	1.64	2.57	1.87	2.53	2.74
Ecuador	1.81	2.88	1.95	2.36	2.54
Estonia	1.47	2.64	1.92	2.24	2.82
Finland	1.01	2.48	1.75	2.62	2.89
Germany	1.25	2.45	1.84	2.61	2.83
Ghana	2.41	2.52	1.90	2.53	2.54
Greece	1.66	2.83	1.71	2.46	2.71
Hungary	1.70	2.44	1.83	2.61	2.69
Iceland	1.26	2.33	1.78	2.62	2.89
India	1.97	2.73	2.03	2.44	2.49
Indonesia	2.46	3.04	2.22	2.00	2.43
Iran	2.33	2.52	2.41	1.79	2.83
Ireland	1.41	2.38	1.91	2.72	2.75
Israel	1.30	2.44	2.11	2.46	2.79
Italy	1.40	2.99	1.72	2.33	2.69
Ivory Coast	2.10	2.55	1.93	2.29	2.67
Japan	1.70	2.54	2.26	2.46	2.82
Kazakhstan	2.26	2.85	2.12	1.96	2.87
Kenya	2.10	2.52	1.97	2.42	2.57
Latvia	1.73	2.64	2.18	2.30	2.54
Malaysia	2.06	2.63	2.10	2.55	2.48
Mexico	1.65	2.69	1.97	2.46	2.59
Mozambique	1.64	2.91	1.93	2.51	2.39
Netherlands	1.63	2.53	1.87	2.60	2.73

Nigeria	2.33	2.64	1.99	2.44	2.46
Peru	1.82	2.77	2.12	2.33	2.56
Poland	1.81	2.71	2.05	2.38	2.43
Portugal	1.51	2.60	1.78	2.49	2.84
Qatar	2.10	2.73	2.20	2.14	2.65
Russia	1.71	2.54	2.06	2.31	2.83
Saudi Arabia	2.20	2.56	2.54	2.08	2.44
Singapore	1.69	2.39	2.03	2.62	2.65
Slovakia	1.77	2.71	2.05	2.36	2.54
South Korea	2.14	2.75	2.14	2.56	2.43
Spain	1.66	2.87	1.78	2.48	2.62
Sri Lanka	2.10	2.57	2.11	2.31	2.70
Sweden	1.05	2.72	1.61	2.68	2.78
Thailand	1.73	2.12	2.51	2.25	2.88
Trinidad and Tobago	1.84	2.32	2.09	2.50	2.61
Turkey	1.66	2.34	2.08	2.59	2.65
UAE	1.96	2.46	2.06	2.46	2.70
Ukraine	1.94	2.62	1.98	2.28	2.83
United Kingdom	1.40	2.21	1.88	2.73	2.83
United States	1.58	2.26	2.02	2.62	2.82
Vietnam	2.03	2.82	2.10	2.51	2.38

Note. The standard errors in these estimates ranged from 0.01 to 0.06 with a mean of 0.03.

Supplementary Table 3. Proportion of variance of city-level metanorms and sample-level (student and non-student) metanorms explained by country.

	physical confrontation	verbal confrontation	social ostracism	gossip	non-action
City-level metanorms	0.93	0.95	0.93	0.91	0.66
Sample-level metanorms	0.91	0.94	0.83	0.90	0.76

Note. R^2 values obtained from one-way ANOVAs of metanorms at city-level (21 cities in 10 countries) or sample-level (student and non-student samples in 31 countries), using country as a single explanatory factor.

Supplementary Table 4. Correlations between the metanorm measures in Supplementary Table 2 and the corresponding measures obtained after exclusion of participants who failed an attention check or found the study difficult to understand.

physical confrontation	verbal confrontation	social ostracism	gossip	non-action
r = .99	r = .99	r = .99	r = .99	r = .98

Note. Correlations are based on n = 57 countries.

Supplementary Table 5. Correlation between metanorms and frequencies of use of informal sanctions.

	Metanorms for			
Item (*M*, *SD*)	physical confrontation	verbal confrontation	social ostracism	gossip
How often do you confront someone who does something inappropriate? (*M* = 3.03, *SD* = 0.22)	.48 [.22,. 67]	.40 [.21,. 57]	-.07 [-.39,. 23]	-.32 [-.54,-. 06]
How often do you avoid someone who does something inappropriate? (*M* = 2.96, *SD* = 0.24)	.19 [-.07,. 47]	-.41 [-.60,-. 15]	.49 [.24,. 66]	.01 [-.25,. 27]
How often do you talk to others about someone who does something inappropriate? (*M* = 3.05, *SD* = 0.23)	-.42 [-.59,-. 23]	-.43 [-.65,-. 22]	-.41 [-.65,-. 14]	.60 [.40,. 76]

Note. Means and standard deviations (in the first column) and correlations, with 95% BCa bootstrapped confidence intervals, are all based on 57 countries.

Supplementary Table 6. How other variables are correlated with metanorm measures estimated separately for non-cooperation and other norm violations.

	physical confrontation in response to		verbal confrontation in response to		social ostracism in response to		gossip in response to	
	non-coop.	insult	non-coop.	out-of-place	non-coop.	out-of-place	non-coop.	out-of-place
Indulgence	-.09<br>[-.38,.18]	-.26<br>[-.45,-.04]	-.05<br>[-.30,.23]	-.17<br>[-.40,.06]	-.27<br>[-.44,-.06]	-.21<br>[-.42,-.00]	.39<br>[.22,.54]	.35<br>[.18,.50]
Power distance	.59<br>[.40,.77]	.66<br>[.48,.77]	.01<br>[-.20,.24]	.39<br>[.20,.55]	.09<br>[-.16,.35]	.37<br>[.14,.54]	-.30<br>[-.51,-.09]	-.45<br>[-.65,-.24]
Individualism	-.58<br>[-.71,-.44]	-.54<br>[-.68,-.37]	-.05<br>[-.37,.27]	-.42<br>[-.65,-.08]	-.08<br>[-.30,.14]	-.38<br>[-.55,-.18]	.25<br>[-.00,.47]	.47<br>[.24,.68]
Individual autonomy	-.41<br>[-.64,-.15]	-.53<br>[-.73,-.27]	-.03<br>[-.28,.22]	-.15<br>[-.36,.05]	-.03<br>[-.30,.23]	-.25<br>[-.49,-.01]	.29<br>[-.01,.62]	.36<br>[.12,.60]
Emancipative moral judgments	-.63<br>[-.78,-.45]	-.74<br>[-.82,-.61]	.10<br>[-.25,.37]	-.25<br>[-.50,.02]	-.22<br>[-.47,.01]	-.54<br>[-.73,-.34]	.50<br>[.29,.70]	.54<br>[.36,.70]
Pro-violence attitudes	.22<br>[.01,.44]	.38<br>[.13,.63]	-.28<br>[-.56,.08]	-.26<br>[-.56,.05]	.14<br>[-.19,.41]	.40<br>[.10,.62]	-.12<br>[-.41,.22]	-.11<br>[-.34,.19]
Tightness	.51<br>[.31,.65]	.43<br>[.16,.62]	-.10<br>[-.30,.11]	-.07<br>[-.28,-.14]	.22<br>[-.03,.46]	.34<br>[.12,.55]	-.17<br>[-.42,.10]	-.16<br>[-.37,.07]
Perceived threat	.22<br>[-.03,.44]	.03<br>[-.24,.31]	.07<br>[-.14,.28]	.21<br>[-.04,.44]	-.18<br>[-.42,.03]	.07<br>[-.17,.30]	.06<br>[-.18,.32]	-.07<br>[-.28,.19]
Pathogen prevalence	.53<br>[.28,.71]	.36<br>[.11,.54]	.15<br>[-.08,.38]	.28<br>[.07,.48]	.02<br>[-.17,.23]	.21<br>[.00,.42]	-.06<br>[-.27,.15]	-.26<br>[-.47,-.03]
Gender equality	-.66<br>[-.78,-.52]	-.66<br>[-.78,-.50]	.05<br>[-.17,.29]	-.12<br>[-.33,.13]	-.42<br>[-.63,-.15]	-.54<br>[-.72,-.30]	.42<br>[.18,.62]	.50<br>[.27,.68]
Median per-capita income	-.62<br>[-.74,-.48]	-.61<br>[-.76,-.40]	-.07<br>[-.38,.18]	-.40<br>[-.62,-.16]	-.21<br>[-.41,.04]	-.33<br>[-.55,-.05]	.39<br>[.21,.55]	.62<br>[.44,.78]

Note. Correlations, with 95% BCa bootstrapped confidence intervals, are based on n = 57 countries, except for indulgence (n = 48), power distance (n = 51), individualism (n = 51), and median income (n = 50).

Supplementary Table 7. Intercorrelations among the predictors used in the study.

	1	2	3	4	5	6	7	8	9	10	11
1. Indulgence	.										
2. Power distance	-.20	.									
3. Individualism	-.01	-.67	.								
4. Autonomy	-.13	-.51	.44	.							
5. Emancipative moral judgments	.15	-.49	.46	.71	.						
6. Pro-violence attitudes	.07	.24	-.22	-.44	-.41	.					
7. Tightness	.03	.30	-.31	-.38	-.43	.39	.				
8. Perceived threats	.22	.10	-.22	.05	-.04	.10	-.04	.			
9. Pathogen prevalence	.10	.53	-.62	-.43	-.39	.31	.33	.43	.		
10. Gender equality	.23	-.57	.42	.39	.51	-.31	-.43	-.13	-.52	.	
11. Median income	.22	-.67	.72	.45	.50	-.13	-.16	-.33	-.65	.52	.

Note. Predictors come from different sources: measures of indulgence, power distance, and individualism are from Hofstede et al.[23]; individual autonomy, emancipative moral judgments, pro-violence attitudes, tightness, and perceived threat were measured in the current study; measures of historical pathogen prevalence are from Murray and Schaller[24]; gender equality is measured by the Global Gender Gap Index from World Economic Forum[25]; median per-capita income measures are from Gallup.[26] Correlations are based on $n = 57$ countries except when involving indulgence, power distance, individualism, or median income. The smallest number of countries is $n = 43$ for the correlation between indulgence and median income.

Supplementary Table 8. How other variables are correlated with metanorm measures for sanctions standardized by the metanorm for non-action.

Predictor	physical confrontation	verbal confrontation	social ostracism	gossip
Indulgence	-.16	-.09	-.15	.33
Power distance	.70	.46	.54	-.02
Individualism	-.63	-.46	-.53	.04
Individual autonomy	-.56	-.29	-.39	.08
Emancipative moral judgments	-.76	-.36	-.64	.20
Pro-violence attitudes	.33	-.12	.35	-.03
Tightness	.43	.01	.40	.07
Perceived threat	.21	.30	.23	.17
Pathogen prevalence	.53	.41	.41	.07
Global gender gap index	-.69	-.21	-.58	.25
Median per-capita income	-.69	-.46	-.51	.23

Note. Predictors come from different sources: measures of indulgence, power distance, and individualism are from Hofstede et al.[23]; individual autonomy, emancipative moral judgments, pro-violence attitudes, tightness, and perceived threat were measured in the current study; measures of historical pathogen prevalence are from Murray and Schaller[24]; gender equality is measured by the Global Gender Gap Index from World Economic Forum[25]; median per-capita income measures are from Gallup.[26] Correlations are based on n = 57 countries, except for indulgence (n = 48), power distance (n = 51), individualism (n = 51), and median income (n = 50).

Supplementary Table 9. Internal consistency (Cronbach's alpha) of country-level appropriateness ratings of responses to different scenarios.

	physical confront. (2 scenarios)	verbal confront. (10 scenarios)	social ostracism (10 scenarios)	gossip (10 scenarios)	non-action (10 scenarios)
Raw country means	.75	.87	.85	.98	.79
Controlled for scenario appropriateness		.92	.91	.98	.84

Note. Based on 57 countries.

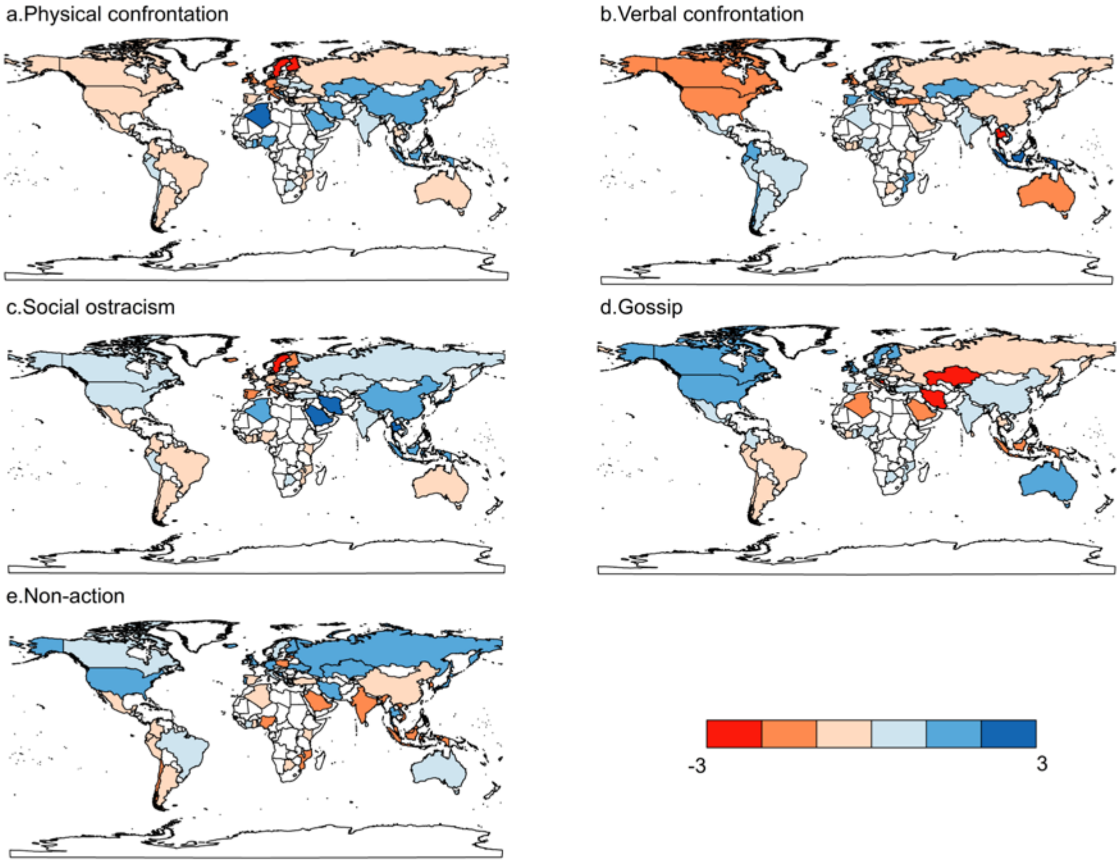

Supplementary Figure 1. Maps depicting country variation in z-scored metanorms for five responses to norm violations. a Physical confrontation. **b** Verbal confrontation. **c** Social ostracism. **d** Gossip. **e** Non-action. The metanorm measures in Supplementary Table 2 are here normalized to have a global mean of 0 and a standard deviation of 1. Every step on the color scale represents 1 standard deviation. The red end of the spectrum indicates country scores below the global mean (i.e., the country rated the response as less appropriate than did the average country), whereas the blue end of the spectrum indicates scores above the global mean. This figure was created using ArcGIS 10.2 software and an original world map retrieved from Global Administrative Areas Version 3.6 (https://gadm.org/), a spatial database of the location of the world's administrative boundaries; using the data to create maps for academic publishing is allowed (https://gadm.org/license.html).
