# Supplementary material for: Perceptions of the appropriate response to norm violation in 57 societies
Source: Nat Commun. 2021 Mar 5;12:1481. doi: 10.1038/s41467-021-21602-9 (PMC7935962; doi:10.1038/s41467-021-21602-9)
Supplement: Supplementary file 3 — Reporting Summary [file 41467_2021_21602_MOESM3_ESM.pdf]

nature research

Corresponding author(s): Kimmo Eriksson

Last updated by author(s): Oct 16, 2020

Reporting Summary

Nature Research wishes to improve the reproducibility of the work that we publish. This form provides structure for consistency and transparency in reporting. For further information on Nature Research policies, see our Editorial Policies and the Editorial Policy Checklist.

Statistics

For all statistical analyses, confirm that the following items are present in the figure legend, table legend, main text, or Methods section.

n/a	Confirmed	
☐	☒	The exact sample size (*n*) for each experimental group/condition, given as a discrete number and unit of measurement
☐	☒	A statement on whether measurements were taken from distinct samples or whether the same sample was measured repeatedly
☐	☒	The statistical test(s) used AND whether they are one- or two-sided<br>*Only common tests should be described solely by name; describe more complex techniques in the Methods section.*
☐	☒	A description of all covariates tested
☐	☒	A description of any assumptions or corrections, such as tests of normality and adjustment for multiple comparisons
☐	☒	A full description of the statistical parameters including central tendency (e.g. means) or other basic estimates (e.g. regression coefficient) AND variation (e.g. standard deviation) or associated estimates of uncertainty (e.g. confidence intervals)
☒	☐	For null hypothesis testing, the test statistic (e.g. *F*, *t*, *r*) with confidence intervals, effect sizes, degrees of freedom and *P* value noted<br>*Give P values as exact values whenever suitable.*
☒	☐	For Bayesian analysis, information on the choice of priors and Markov chain Monte Carlo settings
☐	☒	For hierarchical and complex designs, identification of the appropriate level for tests and full reporting of outcomes
☐	☒	Estimates of effect sizes (e.g. Cohen's *d*, Pearson's *r*), indicating how they were calculated

Our web collection on statistics for biologists contains articles on many of the points above.

Software and code

Policy information about availability of computer code

Data collection	Data were collected using the survey platform Qualtrics (in the version April 2019-January 2020).
Data analysis	Data were analyzed using SPSS v ersion 26.0. The SPSS syntax is described in a document as the OSF site where the data and materials are found.

For manuscripts utilizing custom algorithms or software that are central to the research but not yet described in published literature, software must be made available to editors and reviewers. We strongly encourage code deposition in a community repository (e.g. GitHub). See the Nature Research guidelines for submitting code & software for further information.

Data

Policy information about availability of data

All manuscripts must include a data availability statement. This statement should provide the following information, where applicable:

- Accession codes, unique identifiers, or web links for publicly available datasets
- A list of figures that have associated raw data
- A description of any restrictions on data availability

Data and materials availability: All data and materials are available at OSF (https://osf.io/pm5kc/), including the raw data underlying Figures 1-3 and SPSS syntax for analyses.

Field-specific reporting

Please select the one below that is the best fit for your research. If you are not sure, read the appropriate sections before making your selection.

☐ Life sciences ☒ Behavioural & social sciences ☐ Ecological, evolutionary & environmental sciences

For a reference copy of the document with all sections, see nature.com/documents/nr-reporting-summary-flat.pdf

Behavioural & social sciences study design

All studies must disclose on these points even when the disclosure is negative.

Study description	A quantitative experimental study was designed with the purpose to examine how the appropriateness of informal sanctions varies across (a) different countries, (b) different forms of sanctions, (c) different norm violations. Using a vignette design, the study manipulated, within each subject, the norm violation (using ten different norm violation scenarios) and the form of sanction (verbal confrontation, social ostracism, gossip, and non-action).
Research sample	By recruiting local researchers to the project, we sampled populations in as many and as geographically diverse countries as we could. This process yielded samples in 57 countries across all world regions. Samples were not representative, see sampling strategy below. For comparability of samples, we set out to collect data from approximately 200 college students in a major city in each country. The sample size goal of 200 was set to match a previous, closely related study in Science (Gelfand et al., 2011). The sample size goal was met in almost all countries, and often surpassed. To assess the robustness of the country-level measures obtained from these samples, we complemented the main sampling strategy in two ways: (a) we collected additional data from non-student samples (or, in two cases, part-time students) in 31 countries; (b) we collected data from two or more student samples located in different cities of each of ten countries. In total we have data from 22,863 participants (students: n = 18,091; non-students: n = 4,772), after excluding a few participants (1.5%) who reported an age under 18. The gender composition was 26.4% males, 52.2% females, and 21.4% unknown. Age: M = 25 years, SD = 9.
Sampling strategy	Convenience samples (participants were recruited using invitations via email, on social media, in class, face to face on campus, using public notices and flyers, and using survey organizations). The sample size of at least 200 students per country was determined to match the prior, closely related, study of Gelfand et al. in Science 2011, where they successfully measured how norms and tightness vary across countries.
Data collection	The survey was translated into 30 different languages, following the usual practice of independent translation and back-translation. The study was conducted anonymously online using Qualtrics, with a few exceptions. Part of the Estonian non-student sample and the Ghanaian student and non-student samples were collected using pen and paper at the university, with animations shown on a big screen (and nobody present except participants and experimenters). Researchers were not blinded to the hypotheses of the study.
Timing	Data were collected between 1 April 2019 and 31 January 2020.
Data exclusions	No exclusions were planned, but we had not foreseen that some students would be less than 18 years old. As the study was meant to target adults, we decided to exclude respondents who stated an age below 18 years, which amounted to 1.5% of the data.
Non-participation	Because recruitment methods included open invitations (e.g., on social media and flyers), we have no data on the number of people who could have participated but chose not to.
Randomization	Participants were not allocated into experimental groups. The survey consisted of multiple scenarios and multiple reactions to each scenario; comparisons are across different scenarios and different reactions to scenarios, as well as across countries.

Reporting for specific materials, systems and methods

We require information from authors about some types of materials, experimental systems and methods used in many studies. Here, indicate whether each material, system or method listed is relevant to your study. If you are not sure if a list item applies to your research, read the appropriate section before selecting a response.

Materials & experimental systems

n/a	Involved in the study	
☒	☐	Antibodies
☒	☐	Eukaryotic cell lines
☒	☐	Palaeontology and archaeology
☒	☐	Animals and other organisms
☐	☒	Human research participants
☒	☐	Clinical data
☒	☐	Dual use research of concern

Methods

n/a	Involved in the study	
☒	☐	ChIP-seq
☒	☐	Flow cytometry
☒	☐	MRI-based neuroimaging

Human research participants

Policy information about studies involving human research participants

Population characteristics	See above.
Recruitment	See above. Participants were invited to participate in an "international study of social norms". It is difficult to see how any self-selection bias could drive our main findings about how appropriateness ratings of reactions to norm violations are consistent across scenarios but vary across different reactions in different countries (e.g., that gossip is more preferred in richer countries than in poorer countries, while the opposite holds for physical confrontation).
Ethics oversight	The local researchers in each country acquired ethical approval as required by local regulations (in some countries, anonymous surveys with no sensitive data are exempt from ethics approval). Full information on organizations that approved the study is provided in the manuscript.

Note that full information on the approval of the study protocol must also be provided in the manuscript.
